# MiR-200b Inhibits Tumor Growth and Chemoresistance via Targeting p70S6K1 in Lung Cancer

**DOI:** 10.3389/fonc.2020.00643

**Published:** 2020-05-06

**Authors:** Hui-Fang Jin, Ju-Feng Wang, Ting-Ting Song, Jun Zhang, Lin Wang

**Affiliations:** ^1^Department of Blood Transfusion, The First Affiliated Hospital of Zhengzhou University, Zhengzhou, China; ^2^Department of Oncology, Henan Cancer Hospital, The Affiliated Cancer Hospital of Zhengzhou University, Zhengzhou, China; ^3^Department of Obstetrics and Gynecology, Xijing Hospital, The Fourth Military Medical University, Xi'an, China; ^4^Department of Thoracic Surgery, Henan Cancer Hospital, The Affiliated Cancer Hospital of Zhengzhou University, Zhengzhou, China; ^5^Academy of Medical Sciences, Zhengzhou University, Zhengzhou, China

**Keywords:** miR-200b, tumor growth, p70S6K1, chemoresistance, lung cancer

## Abstract

Downregulation of microRNA-200b (miR-200b) has been identified in a range of cancers, yet the specific mechanisms whereby it influences lung cancer growth require further exploration. We determined that lung cancer patient tumor samples exhibit decreased miR-200b expression, and we further found this miRNA to inhibit tumor growth via interfering with ERK1/2 and AKT signaling, targeting p70S6K1 to suppress HIF-1α expression. This miRNA further rendered H1299 cells more sensitive to cisplatin while impairing their proliferative and invasive potential through its ability to target and inhibit the activity of p70S6K1. These results were further confirmed in a murine xenograft model in which miR-200b also inhibited the growth of tumor and suppressed p70S6K1, p-AKT, p-ERK1/2, and HIF-1α expression. These findings clearly demonstrate a role for miR-200b in suppressing lung cancer development, making it a potentially relevant target for future diagnostic and therapeutic interventions.

## Background

Lung cancers, of which up to 85% are non-small cell lung cancers (NSCLCs), remain one of the deadliest forms of cancer globally (1), with most NSCLC patients not being diagnosed until the disease is relatively advanced, resulting in almost 9 in 10 patients dying due to complications of tumor progression and metastasis (2). Indeed, this disease has just a 17% 5-year survival rate (3, 4). At present, patients with stage II or III NSCLC are typically treated through a combination of surgical tumor resection and cisplatin-based chemotherapy. Once NSCLC has metastasized, however, it tends to not be curable, as the tumors often exhibit intrinsic or acquired chemoresistance (5, 6). The specific mechanisms governing the development and progression of NSCLC remain to be completely characterized.

MicroRNAs (miRNAs) are short RNA molecules of roughly 22 nucleotides in length that lack coding potential (7). These miRNAs are known to regulate cancer cell proliferation, survival, and differentiation through control of particular signaling pathways within the cells (8). The miR-200 family is a group of miRNAs with an overlapping set of target genes and an identical seed sequence, with these miRNAs having been implicated in regulation of epithelial-to-mesenchymal transition (EMT) in tumor cells (9). Many cancers have been found to exhibit abnormal expression of miR-200 family miRNAs, as in the cases of gastric (10, 11), breast (12), bladder (13), and lung (14) cancers. Many miR-200b target genes have been identified at present, such as VEGFR2, CDK2, KRAS, ZEB2, USP25, and UBQLN1 (15–20). Herein, we identify a novel role for miR-200b as an inhibitor of lung cancer growth and chemoresistance.

Herein, we were able to detect reduced miR-200b expression in tumors of human patients with lung cancer. We further explored the role of miR-200b in the growth and functionality of these lung cancer cells, identifying its target genes and exploring the effects of overexpression of this miRNA on lung cancer cell growth and chemoresistance. Together, these findings offer an opportunity to gain new insight into the mechanisms whereby lung cancer develops and progresses, potentially highlighting novel future therapeutic avenues for intervention.

## Methods

### Clinical Specimens

A total of 56 paired normal and lung cancer tissue samples were collected at the First Affiliated Hospital of Zhengzhou University. Samples were snap frozen following surgical resection prior to analysis, with pathologists conducting histological analyses. All patients provided informed consent, and the institutional review board and ethics committees of Zhengzhou University approved this study.

### Cells and Reagents

H1299 lung cancer cells were from American Type Culture Collection. Antibodies specific for p70S6K1, p-AKT, AKT, p-ERK1/2, and ERK1/2 were from Cell Signaling Technology (MA, USA), while those specific for GAPDH were fromBioworld Technology (GA, USA). Growth factor-reduced Matrigel came from BD Biosciences (MA, USA).

### RNA Analysis

Trizol (Invitrogen, CA, USA) was used to extract total RNA based on provided directions, after which qPCR was used to measure miR-200b expression with U6 for normalization. For p70S6K1 expression measurments, GAPDH was used for normalization. A SYBR mix (Takara, Dalian, China) was used for all reactions, with expression changes calculated based on the 2^−ΔΔCt^ approach.

### Immunoblotting

RIPA buffer containing protease inhibitors was used to lyse cells, and protein amounts were assessed via BCA assay (Beyotime, Jiangsu, China). Protein was separated by SDS-PAGE, transferred onto PVDF membranes (Roche, Switzerland), and assessed by Western blotting based on provided directions, using an ECL Detection System (Thermo Scientific, USA) to measure protein levels.

### Cell Proliferation Assay

Indicated miR-NC and miR-200b stable cell lines were plated for 2 × 10^3^ cells per well. To evaluate the proliferation activity of miR-200b in lung cancer cells, according to the manufacturer's instruction, cell proliferation rate was analyzed with CCK-8 kit (Dojindo Laboratories, Japan).

### *In vitro* Invasion Assay

Cellular invasion was determined with 24-well BD Matrigel invasion chambers (BD Biosciences, Cowley, UK) based on provided directions. Following a 16–24 h invasion period, cells that had not become invasive were removed via swabbing, and remaining cells were fixed in 3% paraformaldehyde prior to 0.1% crystal violet staining. Three independent fields were imaged, and OD570 was measured via microplate reader for triplicate samples.

### Dual-Luciferase Reporter Assay

The region of the p70S6K1 3′-UTR containing the putative site for miR-200b binding was amplified and inserted in the pMIR-REPORTER vector (Ambion, CA, USA). Then, 24 h following transfection with these constructs, a Dual Luciferase Reporter Assay System (Promega, WI, USA) was used to assess luciferase activity. Experiments were repeated three times.

### Chemosensitivity Array

A total of 4,000 cells were added in each well of a 96-well plate, and then after 24 h cisplatin (Sigma-Aldrich, MO, USA) was added after fresh preparation, for a final 1–40 μM concentration. After a further 48 h incubation, CCK-8 assay was used to assess viability.

### Flow Cytometry

Apoptosis was assessed via staining cells with PE-Annexin V and propidium iodide (BD Pharmingen), followed by flow cytometry (FACS Canto II, BD Biosciences) analysis, with FlowJo used for data analyses.

### *In vivo* Tumor Growth

Male nude mice (BALB/c-null, 6-week-old) from Shanghai Laboratory Animal Center (Chinese Academy of Sciences, Shanghai, China) were used for all studies. Animals received a subcutaneous posterior flank injection with 5 × 10^6^ tumor cells in a 150 uL total volume in serum-free RPMI-1640. Tumor growth was measured using calipers, and volume was determined based on the formula: volume = 0.5 × (Length × Width^2^). After 28 days of tumor growth, animals were euthanized and tumors were isolated for weighing and western blotting. The Committee of Laboratory Animal Experimentation of Zhengzhou University approved all animal studies.

### Statistical Analysis

GraphPad Prism 5 (CA, USA) was used for all analyses of triplicate experiments. Spearman's rank test was used to assess correlations between the expression of miR-200b and p70S6K1. Other results were compared via *t*-tests, with *P* < 0.05 as the significance threshold.

## Results

### Lung Tumors Exhibit Reduced miR-200b Expression

**Figure 1:**
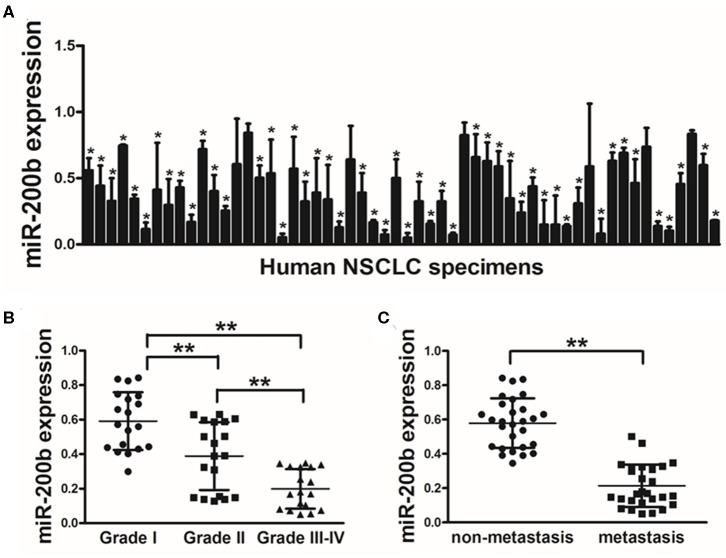
Lung tumors express reduced miR-200b levels. **(A)** Relative miR-200b expression levels were analyzed by Real-time RT-PCR in 56 pairs of lung tumor and adjacent healthy tissue, U6 RNA levels were used as an internal control; **(B)** Relative expression levels of miR-200b in cancer tissues at Grades I, II, and III-IV. **(C)** Expression of miR-200b was significantly lower in NSCLC patients with lymph node metastasis. Data are means ± SD. *P < 0.05; **P < 0.01.

In this study, we began by measuring miR-200b expression via qRT-PCR in lung tumors and matched healthy control tissue samples (56 pairs), revealing significantly lower levels of this tumor in the tumor tissue specimens (Figure 1A). Tumors of WHO Grade III-IV also exhibited significantly reduced miR-200b leaves as compared with Grade I-II tumors, consistent with reduced expression of this miRNA in later stage disease (Figure 1B). We similarly found that there was significantly reduced miR-200b expression in NSCLC samples from patients with lymph node involvement than from those without such involvement (Figure 1C). These results thus suggest that reduced miR-200b expression may be a biomarker of advanced lung cancer progression.

### miR-200b Overexpression Impaired Lung Cancer Cell Proliferation and Invasion

**Figure 2:**
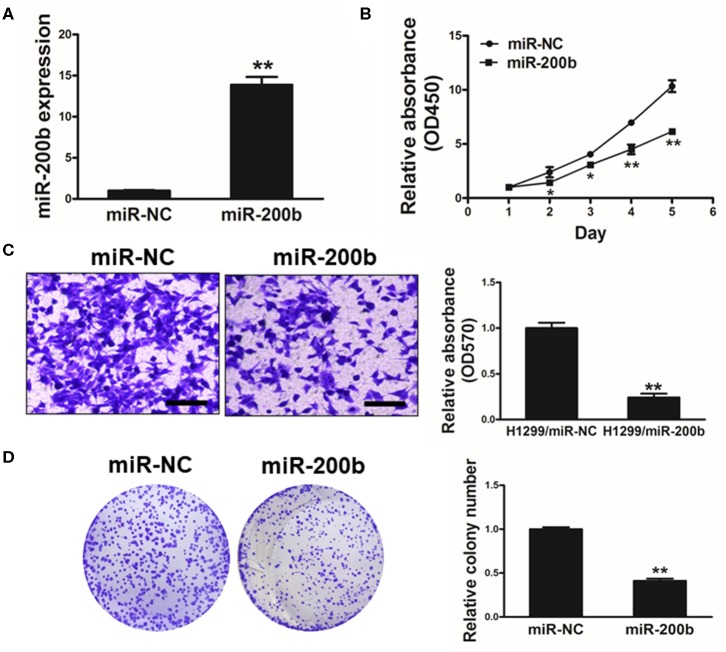
miR-200b expression impairs lung cancer cell proliferation and invasion. **(A)** Relative expression levels of miR-200b in H1299 stable cell lines were determined by real-time RT-PCR; **(B)** Cells were plated in 96-well plates, and cell proliferation was determined using Cell Counting Kit-8 (CCK-8) by detecting the absorbance at 450 nm at indicated time points; **(C)** Migration assay of cells were performed as previously described. miR-200b suppressed H1299 cell invasion. **(D)** Overexpression of miR-200b interfered with H1299 cell colony formation. Data are means ± SD. *P < 0.05; **P < 0.01.

To investigate the function of miR-200b in lung cancer, we next generated H1299 lung cancer cells stably overexpressing miR-200b or NC control for *in vitro* analyses (Figure 2A). Using these cells, we found that miR-200b overexpression was associated with significantly reduced cell growth (Figure 2B).

We further investigated how changes in expression of this miRNA influenced colony formation and invasive activity in lung cancer cells, revealing that miR-200b overexpression significantly impaired both of these processes that are normally pronounced in lung cancer cells (Figures 2C,D). These findings therefore indicated that overexpressing miR-200b can impair the ability of lung cancer cells to proliferate and become invasive.

### miR-200b Targets p70S6K1 to Disrupt AKT and ERK1/2 Signaling

**Figure 3:**
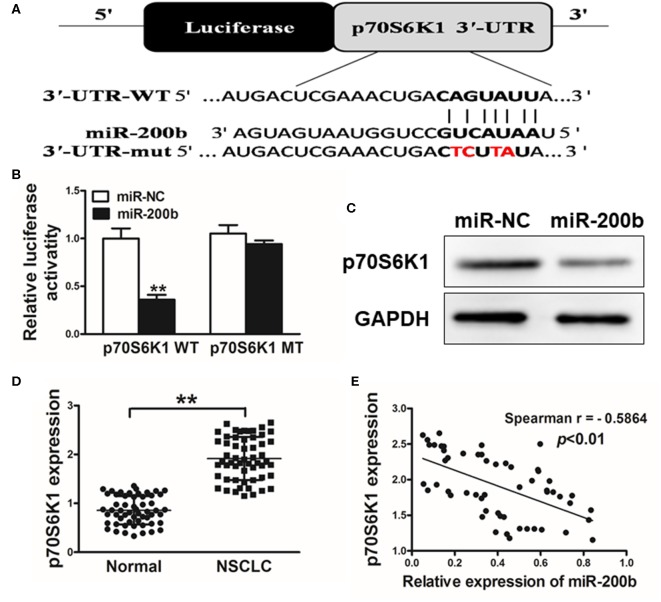
miR-200b targets p70S6K1 in lung cancer. **(A)** The complementary pairings of miR-200b with p70S6K1 wild-type (WT) and mutant (mut) 3′-UTR reporter constructs are shown. The mutant nucleotides of the p70S6K1 3′-UTR region were labeled in red; **(B)** H1299 cells were co-transfected with the reporter constructs containing the WT or mut p70S6K1 3′-UTR, miR-NC or miR-200b and pRL-TK plasmids. The luciferase activities were analyzed 24 h after the transfection. **(C)** Western blotting confirmed reduced p70S6K1 expression following miR-200b overexpression. **(D)** qRT-PCR was used to assess p70S6K1 expression in human tissue samples. **(E)** Spearman's correlations between p70S6K1 and miR-200b in human samples. Data are means ± SD. **P < 0.01.

We next utilized TargetScan (www.targetscan.org) as a means of identifying putative mIR-200b targets, revealing a potential miR-200b binding site in the p70S6K1 3′-UTR (Figure 3A). We next used luciferase reporters bearing either the WT or mutated (MUT) version of this possible miR-200b binding sequence in the pMIR-REPORTER vector. These vectors were transfected into H1299 cells, revealing that miR-200b was able to significantly repress WT but not MUT reporter luciferase activity (Figure 3B). We further confirmed that miR-200b overexpressing cells exhibited reduced p70S6K1 protein levels (Figure 3C). Similarly, we found that tumor tissues from lung cancer patients exhibited significantly increased p70S6K1 relative to normal control samples (Figure 3D). Consistent with this, we found p70S6K1 and miR-200b expression to be negatively correlated in primary lung cancer samples (Figure 3E; Spearman's *r* = −0.5864).

**Figure 4:**
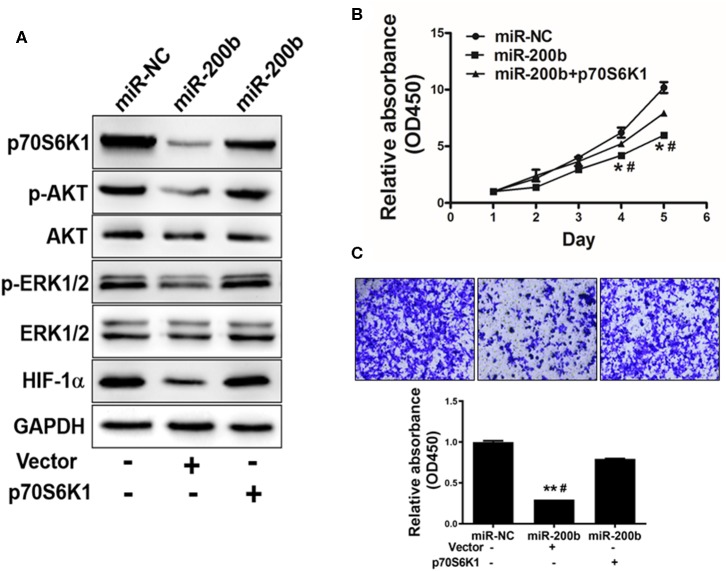
miR-200b suppresses cell proliferation and AKT/ERK1/2 signaling via suppression of p70S6K1. **(A)** Cells overexpressing miR-200b exhibited reduced AKT and ERK1/2 phosphorylation, whereas total levels of these proteins did not change. When p70S6K1 was overexpressed, this normalized the levels of p-AKT, p-ERK1/2, and HIF-1α in cells overexpressing miR-200b. **(B,C)** Elevated miR-200b expression interfered with cellular proliferation and colony formation, whereas p70S6K1 overexpression rescued these phenotypes. Data are means ± SD. *P < 0.05, **P < 0.01 vs. control. ^#^*P* < 0.05 vs. treatment with miR-200b and p70S6K1.

One key mechanism downstream of p70S6K1 is activation of PI3K/AKT and MAPK/ERK signaling, which play key roles in inducing tumor cell growth and induction of genes such as hypoxia-inducible factor-1α (HIF-1α). We found that cells overexpressing miR-200b exhibited markedly reduced p-AKT, p-ERK1/2, and HIF-1α levels relative to controls, whereas total AKT and ERK1/2 levels were unaffected (Figure 4A). To assess whether this effect was dependent upon p70S6K1, we overexpressed p70S6K1 in H1299 cells overexpressing miR-200b or miR-NC (for 48 h using the pCMV-p70S6K1 vector), revealing that this p70S6K1 overexpression was able to reverse this effect, increasing p-AKT, p-ERK1/2, and HIF-1α levels in miR-200b overexpressing cells. This thus indicated that miR-200b targets p70S6K1 to inhibit AKT and ERK signaling.

### miR-200b Inhibits p70S6K1 to Suppress Cell Proliferation, Invasion and Chemoresistance

We next assessed the effects of miR-200b overexpression on H1299 cell functionality in a p70S6K1-dependent manner, revealing that overexpression of p70S6K1 in miR-200b-overexpressing cells restored their ability to proliferate and become invasive (Figures 4B,C).

**Figure 5:**
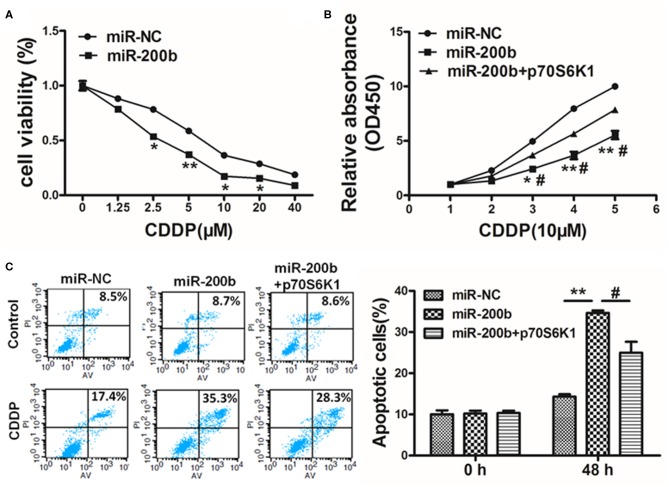
MiR-200b inhibits p70S6K1 to increase lung cancer cell cisplatin sensitivity. **(A)** H1299 cells stably expressing miR-NC or miR-200b were treated with different concentrations of cisplatin for 48 h, and cell viability was analyzed using CCK-8 assay; **(B)** H1299 cells stably expressing miR-NC, miR-200b, or miR-200b in combination with p70S6K1 overexpression were treated with 10 μM of cisplatin for indicated time points. Cell viability was analyzed by CCK-8 assay; **(C)** Cell apoptosis was analyzed by flow cytometry. Data are means ± SD. *P < 0.05, **P < 0.01 vs. control. ^#^*P* < 0.05 vs. treatment with miR-200b and p70S6K1.

Cisplatin resistance is a common cause of failed lung cancer chemotherapy, making it vital that novel means of increasing chemosensitivity be defined. Herein we found that miR-200b overexpression was linked to markedly elevated cisplatin chemosensitivity (Figure 5A). However, when p70S6K1 was overexpressed in these miR-200b overexpressing cells, this reversed their increased sensitivity to cisplatin (10 μM) as determined via CCK-8 assay (Figure 5B). To confirm the relevance of miR-200b and p70S6K1 to cisplatin-induced tumor cell apoptosis, rates of apoptotic death were assessed via flow cytometry, revealing that together miR-200b and cisplatin led to markedly elevated apoptosis rates, while additional p70S6K1 overexpression reversed the effect (Figure 5C). Together these findings suggested that miR-200b thus increases lung cancer cisplatin chemosensitivity, at least in part via targeting of p70S6K1.

### miR-200b Inhibits *in vivo* Tumor Growth

**Figure 6:**
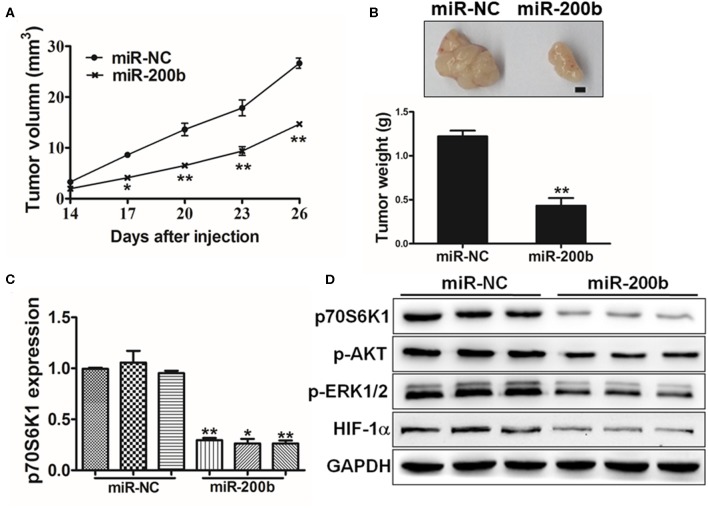
MiR-200b inhibits *in vivo* tumor growth. **(A,B)** Male nude BALB/c mice were injected using 5 × 10^6^ H1299 cells expressing elevated levels of either miR-200b or NC control. The volumes of tumors were monitored for 28 days, after which mice were sacrificed and tumors were weighed, revealing reduced tumor volume and weight in the context of miR-200b overexpression. **(C)** p70S6K1 mRNA levels in tumors overexpressing miR-200b were reduced relative to controls. **(D)** Reduced levels of p70S6K1, p-AKT, p-ERK1/2, and HIF-1α were evident in tumors overexpressing miR-200b relative to controls. Data are means ± SD. *P < 0.05; **P < 0.01.

To assess the importance of miR-200b *in vivo* in the context of tumor progression, we next injected mice with H1299 cells overexpressing either miR-200b or NC control and monitored tumor growth in these animals. Consistent with *in vitro* findings, miR-NC tumors grew significantly larger than miR-200b-overexpressing tumors (Figure 6A), and weighted significantly more (Figure 6B). In addition, the miR-200b-overexpressing tumors had reduced p70S6K1 levels relative to NC controls (Figures 6C,D). Furthermore, miR-200b was associated with reduced p-AKT, p-ERK1/2, and HIF-1α in excised tumor samples (Figure 6D). These results thus confirmed that miR-200b can target p70S6K1 and disrupt lung tumor growth *in vivo*.

## Discussion

Herein we gained novel insights into the role of miR-200b in lung cancer owing to its ability to suppress p70S6K1 expression. miRNAs are known to regulate cancer progression, and many studies have identified links between miRNA expression and patient clinical outcomes. For example, miR-33a-5p has been found to promote lung adenocarcinoma chemosensitivity to celastrol owing to its ability to regulate the activity of mTOR (21). In addition, the upregulation of miR-93-5p was shown to be associated with enhanced NSCLC proliferation and poorer patient outcomes (22). Indeed, many studies have shown miRNAs to play assorted roles in the regulation of lung cancer cell proliferation and chemoresistance.

Herein we observed markedly decreased miR-200b levels in lung cancer tumor samples relative to healthy controls, consistent with previous results exhibiting reduced levels of this miRNA in other tumor types. For example, miR-200b can target FN1 to regulate EMT in breast cancer cells exhibiting chemoresistance (23), and it can similarly suppress the growth of esophageal squamous carcinoma cells, inducing their cell cycle arrest (24). This thus suggests that miR-200b can regulate target gene expression and cancer progression in multiple contexts. In this study, we found that overexpression of miR-200b was sufficient to inhibit lung cancer cell proliferation, invasion, and colony formation. The miR-200 family is composed of 5 miRNA sequences: miR-200a, miR-200b, miR-200c, miR-141, and miR-429, which through the regulation of epithelial to mesenchymal transition (EMT) and cell proliferation to influence tumor growth in various cancers. We also tested miR-200a and miR-200c in lung cancer tissues, there are also downregulated in lung cancer tissues compared with normal tissues. Under this scope, we will conduct further investigation to evaluate their functions in the future, then make sure role of miR-200 family in lung cancer.

P70S6K1 is a gene capable of being regulated via mTOR signaling (25–27), and which is linked to vital cellular processes such as growth, maintenance of glucose levels, and protein synthesis (25). Herein we determined that this p70S6K1 oncogene was a direct miR-200b target, wih an expression level negatively correlated with that of this miRNA in primary human samples. We additionally determined that miR-200b was able to suppress the activation of AKT and ERK1/2 as well as the expression of HIF-1α through its ability to target p70S6K1. These results together thus offer novel evidence in support of a model wherein miR-200b can suppress the growth of cells by inhibiting p70S6K1. While informative, these results do not preclude the possibility that the ability of miR-200b to target other genes also has an impact on lung cancer cell growth, and as such future studies exploring the clinical relevance of additional miR-200b targets are warranted.

Together these results indicate that miR-200b is capable of directly targeting p70S6K1, thereby suppressing lung cancer cell proliferation and invasion, suggesting that this miR-200b/p70S6K1 pathway could represent a novel mechanism governing tumor metastasis and may also serve as a valuable prognostic marker of disease progression.

## Conclusions

In summary, our present investigation suggested that miR-200b functions as a tumor suppressor, as well as sensitizes lung cancer cells to CDDP treatment in an p70S6K1-dependent manner. Given its evident ability to impact metastasis and chemoresistance, the therapeutic value of miR-200b modulation is worthy of future exploration through experimental and clinical validation efforts.

## Data Availability Statement

The raw data supporting the conclusions of this article will be made available by the authors, without undue reservation, to any qualified researcher.

## Ethics Statement

The studies involving human participants were reviewed and approved by Ethics Committee of Zhengzhou University. The patients/participants provided their written informed consent to participate in this study. The animal study was reviewed and approved by Ethics Committee of Zhengzhou University.

## Author Contributions

H-FJ and LW designed the study, analyzed data and drafted the manuscript. J-FW and T-TS participated in the manuscript preparation. H-FJ, LW, J-FW, T-TS, and JZ carried out the experiments *in vitro* and *in vivo*. All authors read and approved the final manuscript.

## Conflict of Interest

The authors declare that the research was conducted in the absence of any commercial or financial relationships that could be construed as a potential conflict of interest.
